# Refractory Hypoglycemia in a Woman With Cirrhosis and a Large Pelvic Mass: A Case Report

**DOI:** 10.7759/cureus.113610

**Published:** 2026-07-29

**Authors:** Michelle E Sherwin, Hloni F Senoamadi, Abraham E Libman, Bhumika Khanna, Roxana Lazarescu

**Affiliations:** 1 Internal Medicine, Xavier University School of Medicine, Oranjestad, ABW; 2 Osteopathic Manipulative Medicine, Touro College of Osteopathic Medicine, New York, USA; 3 Internal Medicine, Wyckoff Heights Medical Center, New York, USA

**Keywords:** cirrhosis, critical care, diagnostic challenge, glycemic variability, hypoglycemia, ovarian neoplasm, paraneoplastic hypoglycemia, point-of-care glucose, refractory hypoglycemia, type 2 diabetes mellitus

## Abstract

Recurrent severe hypoglycemia is a life-threatening condition that may cause altered mental status, metabolic encephalopathy, and loss of protective airway reflexes, particularly in patients with multiple comorbidities and recent changes in glucose-lowering therapy. We report a woman in her 60s with hypertension, chronic obstructive pulmonary disease requiring home oxygen at 2 L/min, atrial fibrillation treated with rivaroxaban, heart failure with reduced ejection fraction of 35%, obesity, and a reported recent diagnosis of type 2 diabetes mellitus. She presented after weakness and a witnessed fall while exiting the shower, without reported loss of consciousness or seizure activity. Although insulin had been prescribed approximately two weeks earlier, she had never obtained or used it. Her initial point-of-care glucose was 69 mg/dL (3.8 mmol/L), followed during hospitalization by recurrent values below 20 mg/dL (below 1.1 mmol/L). Despite repeated dextrose administration, the patient developed progressive somnolence and impaired airway protection requiring endotracheal intubation and intensive care. Management included an initial continuous 10% dextrose infusion, subsequent 5% or 10% dextrose infusions, and repeated 50% dextrose boluses. Head computed tomography (CT) showed no acute intracranial abnormality. Chest and abdominal imaging demonstrated cardiomegaly, pleural effusions, ascites, anasarca, and cirrhotic liver morphology. Pelvic CT and ultrasonography identified a 16.2 × 12.3 × 11.9 cm left adnexal mass concerning a neoplasm. Cancer antigen 125, inhibin A, inhibin B, and insulin-like growth factor binding protein 2 were elevated; however, these findings were nonspecific. Magnetic resonance imaging (MRI) was not completed because the patient declined conventional MRI owing to severe claustrophobia. No biopsy, resection, or histopathologic diagnosis was obtained during hospitalization; the available records documented plans for outpatient open MRI and gynecologic-oncology evaluation but did not specify a separate reason for deferring tissue diagnosis. A complete biochemical critical sample during hypoglycemia, including paired plasma glucose, insulin, C-peptide, proinsulin, beta-hydroxybutyrate, cortisol, screening for insulin secretagogues, and insulin-like growth factor measurements, could not be confirmed, and the available records did not explain why it was not obtained during a qualifying episode. The hypoglycemia was therefore considered likely multifactorial. Poor oral intake, critical illness, and impaired hepatic glycogen storage and gluconeogenesis from cirrhosis were plausible contributors. A tumor-related mechanism remained possible but was not biochemically or pathologically confirmed. The patient was ultimately transitioned from intravenous dextrose to oral glucose supplementation, experienced her final documented hypoglycemic episode on hospital day 34, and was discharged on hospital day 36 for outpatient primary care, endocrinology, hepatology, and gynecologic-oncology evaluation.

## Introduction

Without prompt recognition and treatment, hypoglycemia can become life-threatening. Hypoglycemia may result from medication side effects, changes in nutritional intake, endocrine disease, critical illness, hepatic or renal dysfunction, infection, or tumor-related mechanisms. Glucose-lowering medications can cause hypoglycemia through several pathways. Exogenous insulin increases peripheral glucose uptake and suppresses hepatic glucose production, whereas sulfonylureas and meglitinides stimulate endogenous insulin secretion [1]. Poor oral intake commonly contributes to hypoglycemia during hospitalization, particularly after admission to an intensive care unit (ICU) because hepatic glycogen stores are depleted without adequate nutritional replacement [2].

Cirrhosis can reduce hepatic glycogen storage, glycogenolysis, and gluconeogenesis, thereby limiting the liver’s ability to maintain glucose concentrations during fasting or illness [3]. Severe bacterial infection may increase glucose utilization while inflammatory cytokines suppress hepatic glucose production [3,4]. Certain antimicrobial agents may also contribute to hypoglycemia. Systemic fluoroquinolones are among the most recognized examples. At the same time, clarithromycin, linezolid, and trimethoprim-sulfamethoxazole have also been associated with hypoglycemic events, particularly in susceptible patients or when combined with glucose-lowering medications [5]. Tumors may cause hypoglycemia through endogenous insulin secretion, as in insulinoma, production of insulin-like growth factors, increased glucose utilization, or a combination of these mechanisms. Insulin and insulin-like growth factor signaling can increase peripheral glucose uptake and suppress hepatic glucose output.

Patients with multiple comorbidities can pose a diagnostic challenge when recurrent hypoglycemia has several plausible contributors and treatment of one condition complicates management of another. Diagnostic assessment may also be affected by prolonged hospitalization, extended intensive care, incomplete past medical history, technical limitations of imaging, and patient-specific barriers to completing recommended studies. Ascites and obesity can obscure internal structures and make it more difficult to characterize malignancies, vascular abnormalities, and hepatic disease. Scanner weight or aperture limits, body habitus, subcutaneous and visceral fat, and inability to maintain the required position may further reduce image quality or prevent completion of an examination [6].

Several of these challenges were present in this case. Poor oral intake, critical illness, cirrhotic liver morphology, active hepatitis C, and a large pelvic mass created competing explanations for hypoglycemia. Heart failure with reduced ejection fraction (HFrEF) limited the volume of intravenous dextrose that could be administered safely. Obesity, ascites, and limited mobility restricted pelvic ultrasonographic visualization, while severe claustrophobia prevented completion of conventional magnetic resonance imaging (MRI). This case, therefore, illustrates the difficulty of determining the cause of prolonged hypoglycemia when several physiologic contributors coexist and neither a complete biochemical critical sample nor tissue diagnosis is available.

## Case presentation

A woman in her 60s presented to the emergency department (ED) after weakness and a witnessed fall while exiting the shower. There was no reported loss of consciousness, seizure activity, urinary incontinence, or tongue biting. Her medical history included hypertension, chronic obstructive pulmonary disease requiring home oxygen at 2 L/min, atrial fibrillation treated with rivaroxaban, HFrEF of 35%, obesity, active smoking with an approximately 30-pack-year history, and a reported recent diagnosis of type 2 diabetes mellitus (T2DM). Approximately two weeks before admission, her primary care clinician had prescribed insulin. It was initially considered a potential explanation for the hypoglycemia; however, the patient had never obtained or administered it. The hemoglobin A1c and plasma glucose criteria used to establish the reported T2DM diagnosis could not be confirmed from the records available to the authors. The diagnosis is therefore presented as a reported recent diagnosis rather than independently confirmed in this report.

Her initial point-of-care glucose was 69 mg/dL (3.8 mmol/L), which was mildly low but did not by itself explain the severity of the subsequent clinical course. During the ED evaluation, she developed progressive somnolence and impaired airway protection and was intubated. The available records did not establish that the initial glucose value alone caused the encephalopathy or document a separate primary respiratory indication for intubation. Other metabolic, hepatic, respiratory, and critical-illness contributors were therefore possible. She was admitted to the ICU, where she initially received a continuous 10% dextrose infusion. Subsequent treatment included 5% and 10% dextrose infusions and intermittent 50% dextrose boluses.

The patient was successfully extubated on hospital day 4 and subsequently transferred from the ICU to the hospital floor. Because of the reported recent diagnosis of T2DM, she was initially placed on a carbohydrate-controlled diet before the absence of insulin exposure and persistence of hypoglycemia were fully clarified. She continued to experience decreased appetite and recurrent hypoglycemia, with breakthrough episodes treated using 50% dextrose boluses. Persistent hypoglycemia and poor oral intake prompted a transition to a regular diet on hospital day 17. Nevertheless, oral intake remained limited, and episodes recurred whenever intravenous dextrose was reduced or discontinued.

The patient received no insulin or other hypoglycemic agents during the 36-day hospitalization. Point-of-care glucose measurements demonstrated substantial variability, ranging from below 20 mg/dL (below 1.1 mmol/L) to 284 mg/dL (15.8 mmol/L). Because of HFrEF, peripheral edema, anasarca, and ascites, glucose-containing intravenous fluids had to be administered cautiously to avoid worsening pulmonary or systemic volume overload. Oral glucose tablets were introduced on hospital day 33. The final documented hypoglycemic episode occurred on hospital day 34, and she remained sufficiently stable for discharge on hospital day 36 after continuous intravenous dextrose was discontinued.

**Figure 1:**
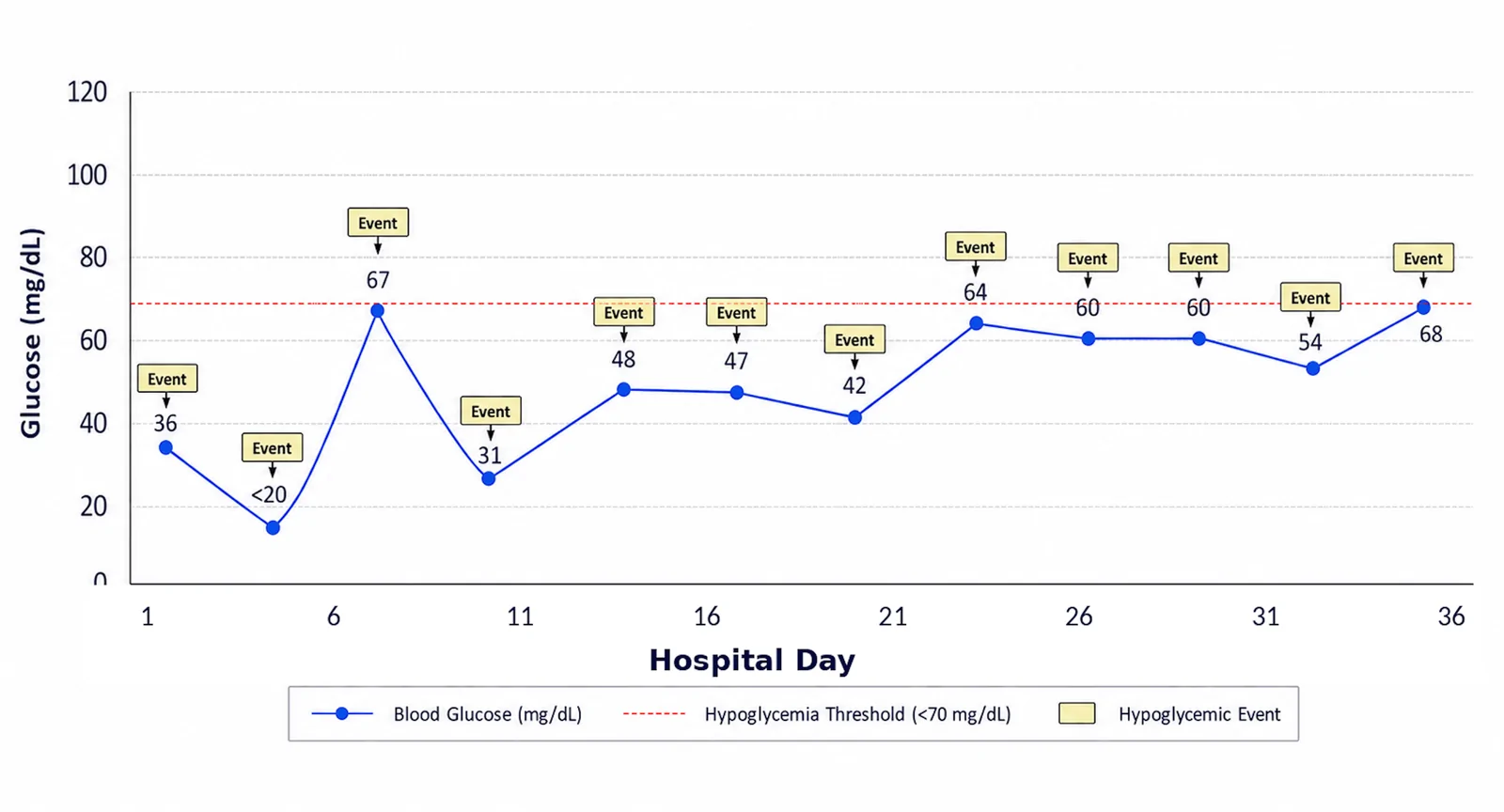
Lowest point-of-care glucose value recorded on each hospital day The lowest point-of-care glucose value was recorded on each day of the 36-day hospitalization. Values below 70 mg/dL (3.9 mmol/L) indicate hypoglycemia. The lowest recorded value was below 20 mg/dL (below 1.1 mmol/L).

**Figure 2:**
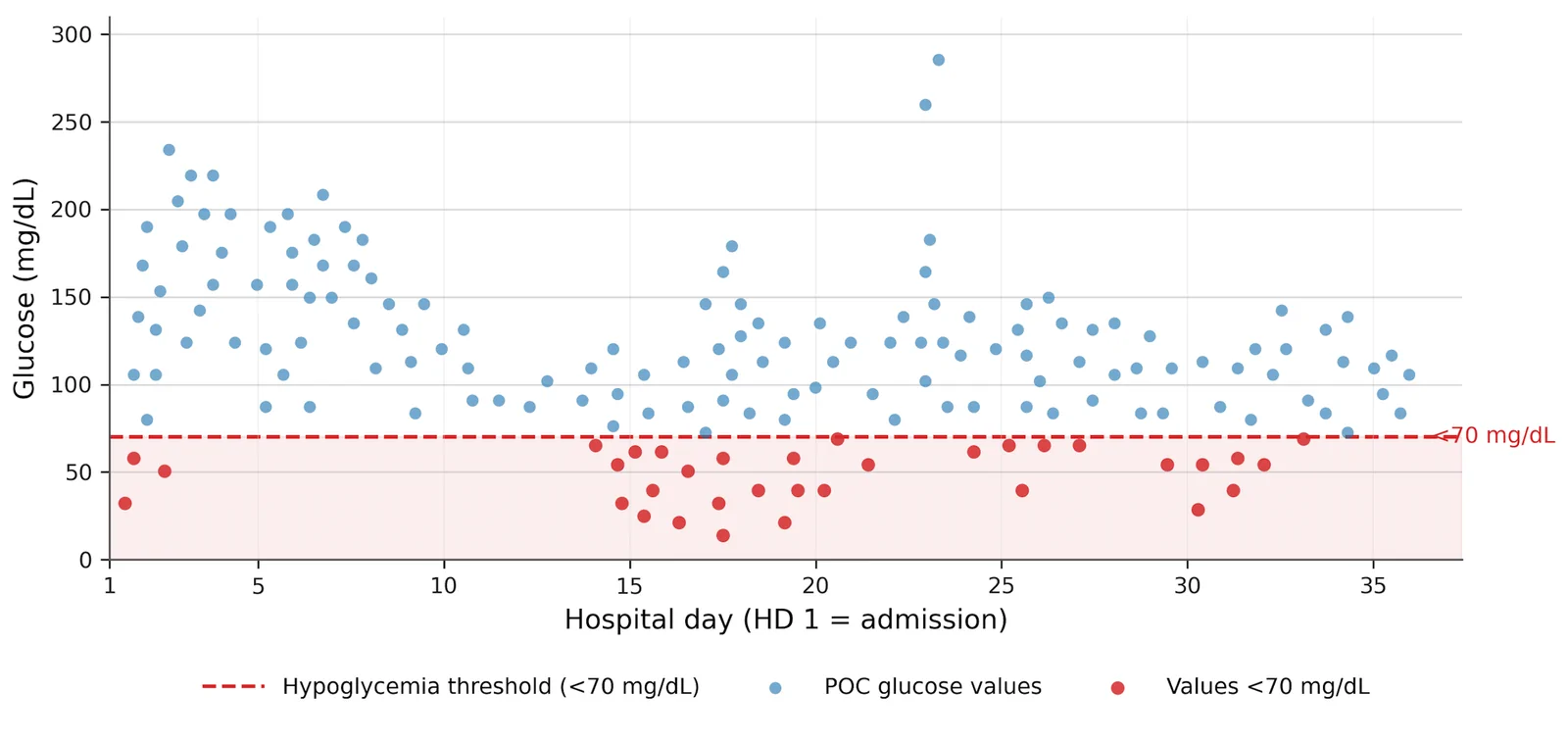
All point-of-care glucose measurements during hospitalization All point-of-care glucose measurements were obtained during the 36-day hospitalization. Each point represents an individual measurement. The dashed line marks the hypoglycemia threshold of 70 mg/dL (3.9 mmol/L). Measurements ranged from below 20 mg/dL (below 1.1 mmol/L) to 284 mg/dL (15.8 mmol/L). HD: hospital day

A complete biochemical critical sample during spontaneous hypoglycemia could not be confirmed from the available records. Clinical notes described the C-peptide concentration as normal and proinsulin as not elevated; however, the exact values, corresponding laboratory plasma glucose concentration, timing relative to treatment, and assay reference ranges were unavailable. Paired plasma glucose, insulin, beta-hydroxybutyrate, cortisol, insulin antibodies, a sulfonylurea/meglitinide screen, insulin-like growth factor I, and insulin-like growth factor II were not documented during a qualifying hypoglycemic episode. The records did not indicate whether the missing components had not been obtained or were simply unavailable for retrospective review, and they did not document a reason for the incomplete evaluation. Accordingly, endogenous hyperinsulinism, adrenal insufficiency, surreptitious exposure to an insulin secretagogue, and non-islet-cell tumor hypoglycemia (NICTH) could not be biochemically confirmed or completely excluded. The lowest point-of-care glucose value recorded on each hospital day is shown in Figure 1. The complete distribution of point-of-care glucose measurements throughout the hospitalization is shown in Figure 2.

Initial chest imaging demonstrated bilateral pulmonary opacities, prompting evaluation for pneumonia because severe bacterial infection can contribute to hypoglycemia. Subsequent clinical findings did not support an active bacterial pulmonary infection: no leukocytosis was documented, the patient remained afebrile, and respiratory symptoms did not persist after extubation. The bilateral opacities were therefore considered more consistent with pulmonary edema in the setting of HFrEF and generalized volume overload. This distinction was clinically important because infection and cardiac congestion could each affect glucose regulation and treatment.

**Figure 3:**
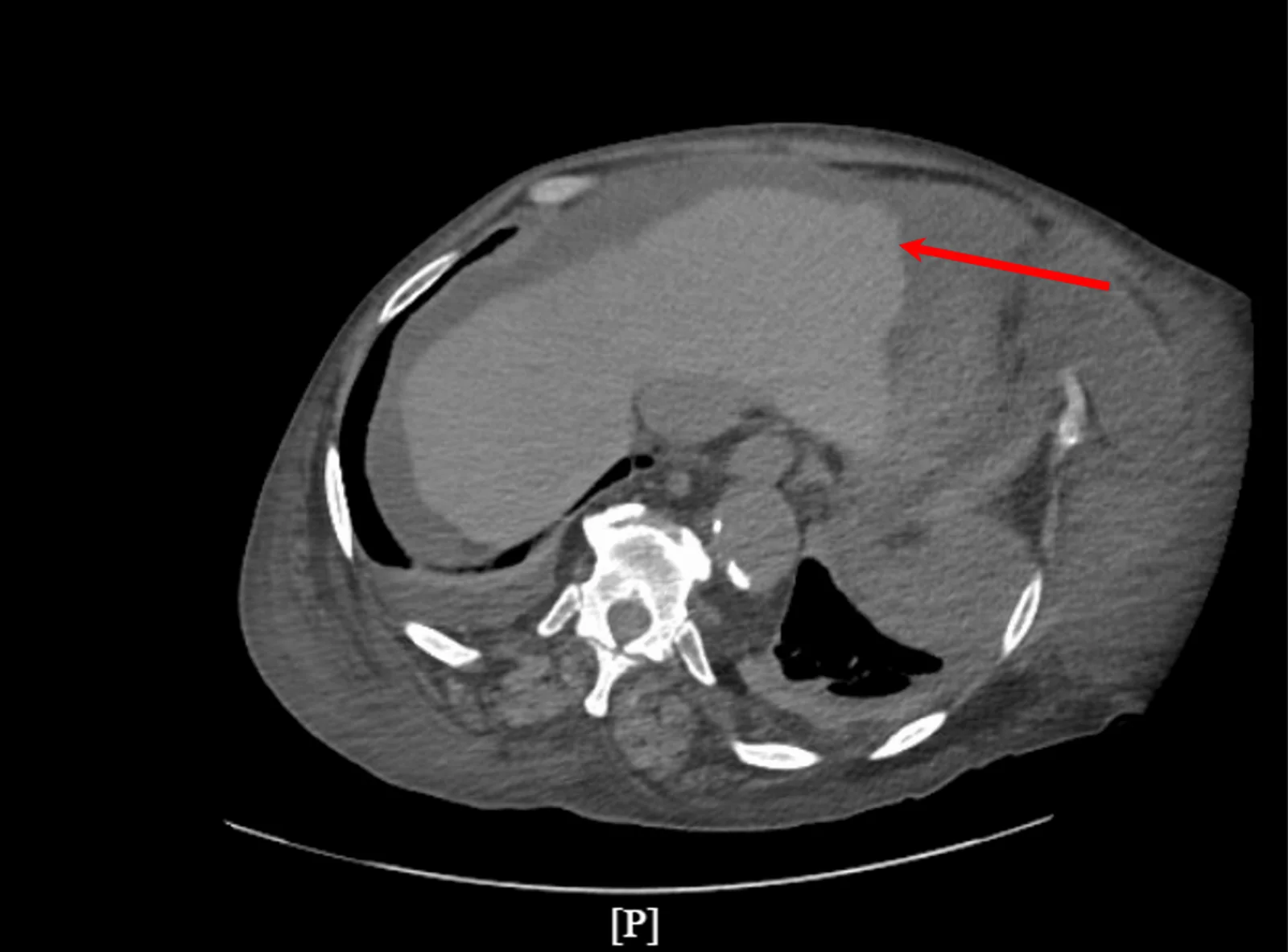
CT of the abdomen demonstrating cirrhotic liver morphology Axial CT image showing a nodular hepatic contour consistent with cirrhotic morphology (arrow) with associated abdominal ascites. CT: computed tomography

**Figure 4:**
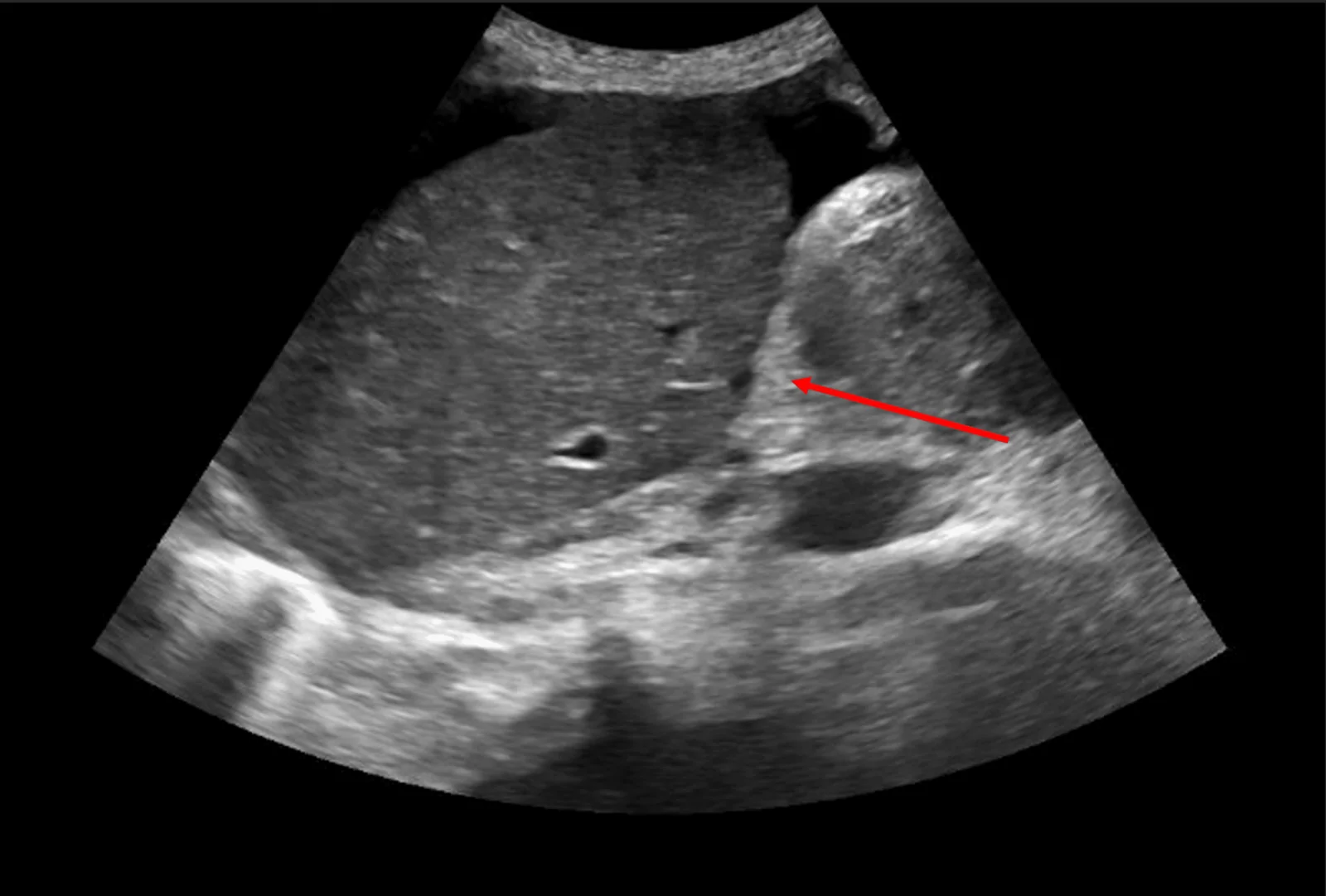
Right upper quadrant US demonstrating cirrhotic liver morphology Right upper quadrant US showing nodular hepatic contour and coarse parenchymal echotexture (arrow), findings concerning for cirrhosis. US: ultrasonography

Abdominal and pelvic computed tomography (CT) on hospital day 1, with repeat CT on hospital day 20, and right upper quadrant ultrasonography (US) on hospital day 10 demonstrated nodular hepatic morphology and coarse parenchymal echotexture consistent with cirrhosis (Figures 3-4). The imaging findings, active hepatitis C infection, poor nutritional intake, and prolonged critical illness all supported hepatic dysfunction as a plausible contributor to impaired glucose regulation.

**Figure 5:**
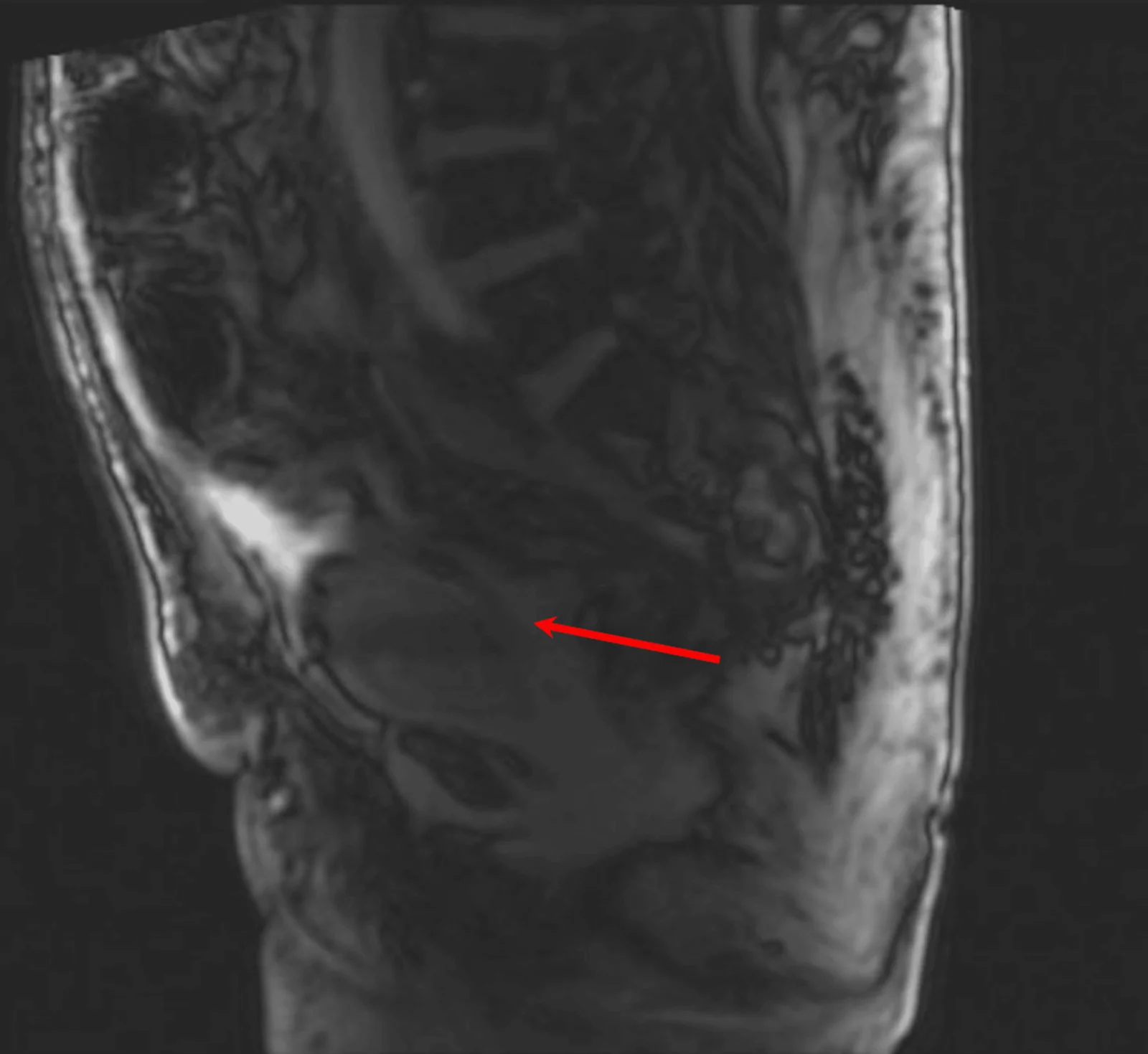
Axial CT of the abdomen and pelvis demonstrating a large pelvic mass Axial CT image showing a large heterogeneous pelvic mass (arrow), abdominal ascites, and anasarca. The organ of origin was indeterminate on CT. CT: computed tomography

**Figure 6:**
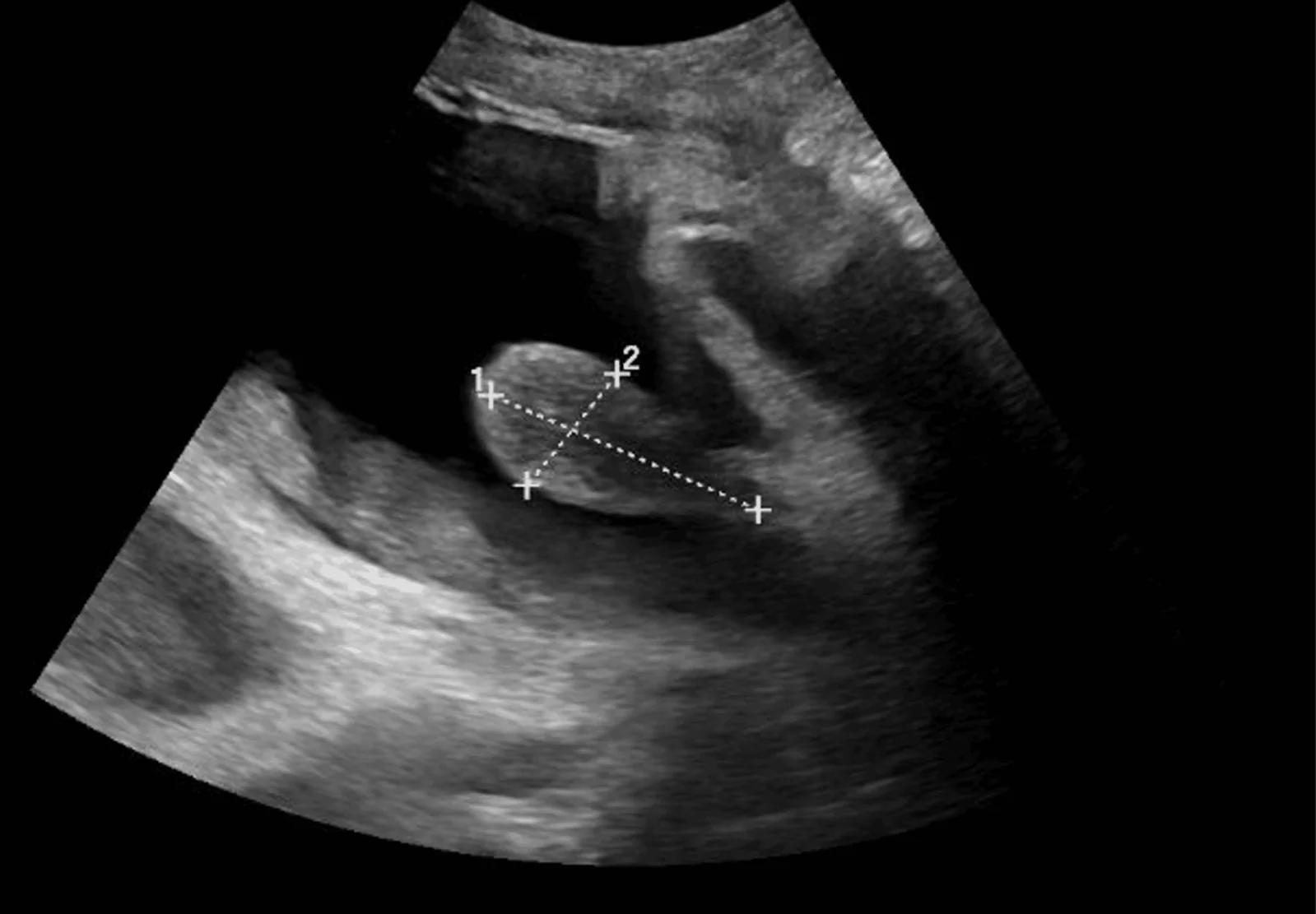
Pelvic US demonstrating a large left adnexal mass Pelvic US showing a 16.2 × 12.3 × 11.9 cm hypoechoic left adnexal mass (arrow). The left ovary was not separately visualized. The findings were concerning for a neoplasm but were not histologically confirmed. US: ultrasonography

Laboratory testing on hospital day 9 demonstrated active hepatitis C infection. Pelvic CT on hospital day 1, with repeat imaging on hospital day 20, demonstrated a large heterogeneous pelvic mass that was initially interpreted as an enlarged heterogeneous uterus (Figure 5). Pelvic US on hospital day 10 instead demonstrated a 16.2 × 12.3 × 11.9 cm hypoechoic left adnexal mass concerning for neoplasm (Figure 6). The uterus measured 5.6 × 4.4 × 2.8 cm and had normal myometrial echotexture. Neither ovary was clearly visualized separately from the mass because of body habitus, ascites, and limited mobility during the examination.

Additional characterization with MRI was recommended because CT and US could not establish the lesion’s organ of origin, histologic type, or stage. The patient declined conventional MRI during hospitalization because of severe claustrophobia but agreed to pursue outpatient open MRI. No biopsy, surgical resection, or histopathologic examination was performed during the admission. The available records documented a plan for outpatient imaging and gynecologic-oncology evaluation but did not specify a separate reason for deferring tissue diagnosis. The lesion therefore remained a suspected left adnexal neoplasm or large pelvic mass rather than a confirmed ovarian tumor. Although the mass provided one possible explanation for the ascites and raised consideration of tumor-related hypoglycemia, neither relationship was definitively established.

The patient’s oral intake improved during the latter portion of the hospitalization, although nutritional intake remained an important consideration in preventing recurrent hypoglycemia. Oral glucose tablets, 4 g up to five times daily as needed, were provided for hypoglycemia rescue. The final documented hypoglycemic episode occurred on hospital day 34, and the glucose concentration at discharge was 133 mg/dL (7.4 mmol/L). She was discharged on hospital day 36 with glucose tablets and referrals for primary care, endocrinology, hepatology, and gynecologic-oncology follow-up. The outpatient plan included confirmation or reconsideration of the reported diabetes diagnosis, continued glucose monitoring, management of hepatitis C and cirrhosis, nutritional assessment, and definitive characterization of the pelvic mass through open MRI and specialist evaluation. Subsequent outpatient records were unavailable; therefore, completion of the planned imaging, tissue diagnosis, endocrine evaluation, and post-discharge glucose outcomes could not be assessed.

## Discussion

This case illustrates prolonged, recurrent severe hypoglycemia during a 36-day hospitalization despite the absence of documented exposure to insulin or another hypoglycemic agent. The principal findings were the coexistence of multiple plausible etiologies, incomplete diagnostic evaluation, and the challenge of balancing glucose replacement with heart failure and generalized volume overload. Because recurrent severe hypoglycemia carries clinically important neurologic and cardiovascular risks, prevention of further episodes remained the immediate priority [7].

Following transfer from the ICU, management continued to require repeated reassessment. The initial carbohydrate-controlled diet reflected the reported recent diagnosis of T2DM rather than an attempt to treat the emerging hypoglycemic disorder. Once the absence of insulin exposure, persistent hypoglycemia, and poor oral intake were clarified, the diet was liberalized on hospital day 17. However, episodes recurred whenever intravenous dextrose was reduced or discontinued. HFrEF, pulmonary congestion, peripheral edema, anasarca, and ascites required cautious administration of glucose-containing fluids to avoid worsening systemic or respiratory congestion, consistent with the need for careful fluid management in patients with heart failure [8]. Oral glucose tablets were introduced late in the hospitalization and were followed by successful discontinuation of continuous intravenous dextrose. This course emphasizes that management involved not only correcting isolated glucose values but also maintaining adequate carbohydrate delivery, improving nutrition, and balancing treatment against the risk of worsening volume overload.

The pelvic mass prompted a broad tumor-marker evaluation. Cancer antigen 125, inhibin A, inhibin B, and insulin-like growth factor binding protein 2 were elevated, whereas cancer antigen 19-9, alpha-fetoprotein, and estradiol were not. Cancer antigen 19-9 was included as part of the broad evaluation despite low suspicion for pancreaticobiliary disease because imaging was limited and the hypoglycemia remained unexplained. These results increased concern for a possible gynecologic neoplasm but did not establish ovarian origin, malignant histology, stage, or a tumor-mediated hypoglycemic syndrome. Cancer antigen 125 is sensitive but nonspecific and may be elevated in ovarian malignancy, ascites, serosal inflammation, and other benign or malignant conditions. Alpha-fetoprotein may support consideration of selected germ-cell tumors, while inhibin A and inhibin B may be associated with sex cord-stromal tumors; estradiol may provide additional context in selected hormone-producing tumors [9]. The mildly elevated insulin-like growth factor binding protein 2 concentration was not a measurement of insulin-like growth factor II and did not confirm NICTH. The available tumor-marker results are summarized in Table 1.

**Table 1:** Selected tumor-marker results obtained during evaluation of the pelvic mass Tumor-marker results obtained throughout the patient's 36-day hospital stay. All reference values listed correspond to normal values for postmenopausal women. These markers are nonspecific and do not establish the histologic origin or malignant nature of the pelvic mass. Insulin-like growth factor binding protein 2 is not equivalent to insulin-like growth factor II and does not confirm NICTH. CA 19-9: cancer antigen 19-9, AFP: alpha-fetoprotein, CA-125: cancer antigen 125, IGFBP-2: insulin-like growth factor binding protein 2, NICTH: non-islet-cell tumor hypoglycemia, pg/mL: picograms per milliliter, ng/mL: nanograms per milliliter, units/mL: units per milliliter

Test	Result	Reference range
CA 19-9	<2 units/mL	<37 units/mL
AFP	<1.8 ng/mL	<10 ng/mL
Inhibin A	10 pg/mL	<2.1 pg/mL
Inhibin B	44 pg/mL	<10 pg/mL
Estradiol	<30 pg/mL	<31 pg/mL
CA-125	301 units/mL	<35 units/mL
IGFBP-2	375 ng/mL	47-350 ng/mL

The overall pattern favored a multifactorial etiology. Poor oral intake reduced exogenous carbohydrate availability and likely depleted hepatic glycogen stores, while prolonged critical illness increased metabolic demand and reduced nutritional reserve. Bacterial pneumonia was considered early because of the bilateral pulmonary opacities, but the patient remained afebrile, no leukocytosis was documented, and respiratory symptoms did not persist after extubation; these findings did not support active bacterial pulmonary infection as the principal cause [4]. Cirrhosis was a more plausible contributor because loss of functioning hepatocyte mass can impair glycogen storage, glycogenolysis, and gluconeogenesis, especially during fasting, poor intake, and systemic illness [3]. Active hepatitis C and imaging findings of nodular hepatic morphology supported chronic liver disease, although hepatic dysfunction could not be isolated from the other contributors. This interpretation is consistent with prior literature describing hypoglycemia in critical illness [2]. Yen et al. found that patients with T2DM and compensated cirrhosis had an adjusted 2.74-fold greater risk of severe hypoglycemia than patients without cirrhosis [3]. Although the diabetes diagnosis in the present case was unconfirmed, that study supports cirrhosis as a biologically plausible contributor. This case differed from medication-associated hypoglycemia because the patient had not obtained the recently prescribed insulin and received no insulin or other hypoglycemic agent during the hospitalization [1,5].

A formal biochemical distinction between insulin-mediated and non-insulin-mediated hypoglycemia was not possible. Clinical notes described the C-peptide concentration as normal and proinsulin as not elevated. Still, the exact values, corresponding laboratory plasma glucose concentration, timing relative to treatment, and assay reference ranges were unavailable. Without paired glucose, insulin, C-peptide, proinsulin, beta-hydroxybutyrate, cortisol, and insulin-secretagogue testing during a qualifying episode, endogenous hyperinsulinism, adrenal insufficiency, medication exposure, and other endocrine causes could not be definitively evaluated. Pancreatic insulinoma, therefore, was not formally excluded, although pancreatic localization would ordinarily be pursued only after a biochemical pattern supporting endogenous hyperinsulinism had been demonstrated. The missing critical sample did not prove a non-insulin-mediated mechanism; rather, it limited the strength of every etiologic conclusion and required cautious interpretation.

Heart failure was relevant both diagnostically and therapeutically. Impaired left ventricular systolic function can increase left-sided filling pressures and pulmonary capillary hydrostatic pressure, allowing fluid to move into the pulmonary interstitium and alveolar spaces and producing pulmonary edema and bilateral radiographic opacities. Dependent regions may be particularly affected because of gravitational variation in perfusion and hydrostatic pressure. Generalized cardiac congestion may also contribute to pleural effusions, peripheral edema, hepatic and systemic venous congestion, anasarca, and ascites [8]. In this patient, the bilateral opacities were ultimately considered more consistent with congestion than pneumonia, and the same volume-overloaded state limited the amount of intravenous dextrose that could be administered safely. The team therefore had to provide sufficient carbohydrates to reduce the risks of neurologic injury, arrhythmia, myocardial ischemia, and other cardiovascular complications while avoiding worsening respiratory or systemic congestion. This tension explains why the transition to oral glucose supplementation was clinically important even though it did not establish the underlying cause.

Definitive characterization of the pelvic lesion was not achieved. CT demonstrated a large heterogeneous pelvic mass, while US localized it to the left adnexal region but could not separately identify the ovary. Conventional MRI was not completed because of severe claustrophobia, a recognized barrier to enclosed imaging studies [10]. No biopsy, resection, or histopathologic diagnosis was obtained during the admission; the available records documented a plan for outpatient open MRI and gynecologic-oncology evaluation but did not specify a separate reason for deferring tissue diagnosis. The lesion therefore remained a suspected left adnexal neoplasm rather than a confirmed ovarian tumor. NICTH has been reported with large fibrous and other mesenchymal tumors and with selected hepatic, epithelial, and ovarian malignancies [11]. Excess or incompletely processed insulin-like growth factor II may activate insulin receptors, increase peripheral glucose uptake, and suppress hepatic glucose production, insulin secretion, lipolysis, and ketogenesis. Rapidly proliferating tumors may also increase glucose utilization and direct glucose through pathways such as the pentose phosphate pathway, which supports biosynthesis and resistance to oxidative stress [12]. Selloua et al. described NICTH in pathologically confirmed high-grade serous ovarian carcinoma with suppressed insulin and C-peptide concentrations and an elevated insulin-like growth factor II/insulin-like growth factor I ratio [11]. In contrast, insulin-like growth factor I, insulin-like growth factor II, and their ratio were not measured in this patient, and no tissue diagnosis was available. The association between the mass and hypoglycemia was therefore biologically plausible but unproven.

The reported recent diagnosis of T2DM also required reassessment. The hemoglobin A1c and plasma glucose criteria used to establish that diagnosis were unavailable, and the patient developed prolonged hypoglycemia without receiving glucose-lowering therapy. Cirrhosis, anemia, altered red-cell survival, recent illness, and nutritional instability may complicate interpretation of hemoglobin A1c and fasting glucose. When cirrhosis and diabetes truly coexist, management is difficult because hepatic dysfunction reduces endogenous glucose reserve while some glucose-lowering medications increase hypoglycemia risk. Oral agents differ in their risk of serious hypoglycemia [13], but this case does not support recommending metformin, a thiazolidinedione, a glucagon-like peptide-1 receptor agonist, or any other specific drug class. Future therapy should be considered only after recurrent hypoglycemia has resolved, the diabetes diagnosis has been confirmed, nutrition and liver disease have stabilized, and cardiac and renal status have been reassessed. If diabetes is confirmed, routine evaluation for renal, retinal, neurologic, and cardiovascular complications should be incorporated into longitudinal care, and nutritional counseling should emphasize avoidance of fasting and inadequate carbohydrate intake. At the same time, the cause of hypoglycemia remains unresolved.

The principal diagnostic lesson is the importance of obtaining a complete critical sample during spontaneous hypoglycemia whenever clinically feasible without delaying urgent treatment. Recommended testing includes paired laboratory plasma glucose, insulin, C-peptide, proinsulin, beta-hydroxybutyrate, and an insulin-secretagogue screen [14]. Cortisol, insulin antibodies, insulin-like growth factor I, and insulin-like growth factor II may be added when the clinical context suggests adrenal, autoimmune, or tumor-related disease. Insulin-like growth factor II-mediated NICTH is reported most often with large mesenchymal or fibrous tumors, although hepatic, epithelial, and ovarian malignancies have also been described [15]. Continued care in this case required endocrinology review of the inpatient glucose pattern and consideration of additional supervised testing; gynecologic-oncology evaluation and open MRI to characterize the pelvic mass and determine whether biopsy or resection was appropriate; hepatology care for active hepatitis C and cirrhotic morphology; and primary care and cardiology follow-up to integrate nutrition, liver disease, heart failure, volume status, and recurrent hypoglycemia.

Ethics and data source

Clinical data were obtained through retrospective review of physician notes, laboratory studies, imaging findings, consulting-physician evaluations, medication-administration records, and discharge documentation. Diagnostic evaluation included serial glucose monitoring, CT, abdominal and pelvic US, laboratory testing, and tumor-marker analysis. Identifiable patient information was removed to protect privacy. Institutional review board approval was not required for this single-patient case report in accordance with institutional policy.

Limitations

This retrospective report is limited by incomplete diagnostic and follow-up data. A complete critical sample during spontaneous severe hypoglycemia could not be confirmed, and the records did not explain why it was not obtained during a qualifying episode. Exact paired plasma glucose, insulin, C-peptide, proinsulin, beta-hydroxybutyrate, cortisol, insulin-antibody, and insulin-secretagogue results were unavailable, and the timing of the reported C-peptide and proinsulin results relative to hypoglycemia and treatment was unclear. Insulin-like growth factor I, insulin-like growth factor II, their ratio, and the criteria underlying the reported T2DM diagnosis were also unavailable.

Conventional MRI was not completed because of severe claustrophobia. No biopsy, resection, or histopathologic diagnosis was obtained during admission; outpatient open imaging and gynecologic-oncology evaluation were planned, but the records did not state a separate reason for deferring tissue diagnosis. US was restricted by body habitus, ascites, and limited mobility. Poor intake, critical illness, cirrhosis, active hepatitis C, heart failure, and volume overload remained confounders, and post-discharge imaging, pathology, endocrine evaluation, glucose outcomes, and follow-up were unavailable. Therefore, this report cannot establish a single cause of hypoglycemia, confirm the mass’s histology, or diagnose NICTH. Because this was a retrospective review, the reasons that certain evaluations were not performed could not always be determined from the available documentation.

## Conclusions

This case demonstrates prolonged, recurrent severe hypoglycemia during a 36-day hospitalization despite no documented exposure to insulin or another hypoglycemic agent. Poor oral intake, prolonged critical illness, cirrhotic liver disease with active hepatitis C, and impaired hepatic glycogen storage, glycogenolysis, and gluconeogenesis were plausible contributors. A large suspected left adnexal neoplasm raised concern for a tumor-related mechanism, but this remained unproven because a complete critical sample, insulin-like growth factor testing, definitive MRI, and tissue diagnosis were unavailable. The etiology was therefore most likely multifactorial.

The prolonged need for intravenous dextrose, inability to administer unrestricted fluids because of heart failure and generalized volume overload, and eventual transition to oral glucose supplementation illustrate the central management challenge. In patients with unexplained recurrent hypoglycemia, clinicians should obtain a complete critical sample during a spontaneous episode whenever feasible without delaying treatment, confirm or reconsider any unconfirmed diabetes diagnosis, and avoid attributing hypoglycemia to a tumor without biochemical and pathologic support. Carbohydrate replacement should be individualized to cardiac and volume status, with oral supplementation used when clinically appropriate. Diagnostic conclusions should remain cautious when definitive biochemical testing, imaging, and histopathology cannot be obtained.
